# Primary Metabolites, Anthocyanins, and Hydrolyzable Tannins in the Pomegranate Fruit

**DOI:** 10.3389/fpls.2019.00620

**Published:** 2019-05-17

**Authors:** Irit Bar-Ya'akov, Li Tian, Rachel Amir, Doron Holland

**Affiliations:** ^1^Unit of Deciduous Fruit Tree Sciences, Newe Ya'ar Research Center, Agricultural Research Organization, Rishon LeZion, Israel; ^2^Department of Plant Sciences, University of California, Davis, Davis, CA, United States; ^3^Laboratory of Plant Metabolism, Department of Plant Science, Migal, Tel Hai College, Qiryat Shmona, Israel

**Keywords:** pomegranate, fruit, lipids, sugars, polyphenols, proteins, organic acids, metabolites

## Abstract

Pomegranate (*Punica granatum* L.) is an important and interesting fruit tree that is cultivated in many parts of the world. In recent years, along with the increase in its cultivation and consumption there has been a dramatic increase in the scientific interest in its biology, methods of cultivation, adaptation to environmental cues and its health-promoting properties. Quite a large proportion of the various metabolites produced in the pomegranate were determined and their content in the bark, roots, leaves, and fruit was reported. Many reviews on polyphenolic compound content, antioxidant activity and health-promoting compounds were published recently. However, only very few recent reports were dedicated to primary metabolites, despite the fact that much work was done on organic acids, sugars, proteins, lipids, and amino acids of the pomegranate fruit. In this review, a special effort was made to present these recent studies and the review is devoted to primary metabolites. The reported data show high variation in the content of primary metabolites within the pomegranate fruit; therefore the data is presented (whenever possible) according to fruit tissues (peel, arils, and seeds), developmental stages of the fruit, environmental and climatic conditions, and genetic background. Most of the data on pomegranate is based on metabolic content and contains no genetic or molecular analysis except for work done on anthocyanins and hydrolyzable tannins. In those cases, gene assignment and genetic control studies were pointed out in the review. The recent publication of the genome sequences from several pomegranate varieties and transcriptomic data from fruits, flowers, and leaves is expected to facilitate the understanding of genetic control of metabolites in pomegranate.

## Introduction

Pomegranate (*Punica granatum* L.) is a fruit tree grown today in a wide range of subtropical and tropical geographical locations spread all over the globe; these locations include many countries in Asia, Europe, South and North America, Africa, and Australia (Holland et al., 2009). Pomegranate is considered a minor fruit and is far from the top of the list of consumed fruits, such as apple, banana, grapes, and citrus; however, it is one of the most interesting fruits in terms of cultural, traditional, and potential therapeutic usage.

The pomegranate fruit is a fleshy berry with a nearly round shape, crowned by a prominent calyx. Its relatively thick peel has an outer colored skin and the fruit's inner structure contains multi-arils chambers separated by membranous walls (Holland et al., 2009). The edible part of the pomegranate fruit, the arils, contains seeds and a special layer of cells (juice cells) that are of epidermal origin and protrude from the outer epidermal cells of the seed (Fahan, 1976; Holland et al., 2009). The external fruit color ranges from yellow, green or pink overlaid with pink to deep red or deep purple. The color of the juicy layer can vary from white to deep red (Holland et al., 2009). Various parts of the pomegranate fruit were traditionally used as treatments against various ailments including stomachaches and bacterial infections (Holland and Bar-Ya'akov, 2014). The traditional usages were strengthened by modern scientific studies focused on health beneficial metabolites and their therapeutic effects and mechanisms of action on human and animal health. These studies were thoroughly reviewed in recent years. Most of the therapeutic effects of the pomegranate fruit were attributed to its secondary and primary metabolites, such as polyphenols, including flavonoids, anthocynains and hydrolizable tannins, fatty acids, and lipids (Seeram et al., 2006a; Lansky and Newman, 2007; Jurenka, 2008; Viuda-Martos et al., 2010; Teixeira da Silva et al., 2013; Holland and Bar-Ya'akov, 2014; Wu and Tian, 2017). These metabolites were found in all fruit parts, including the fruit peel (ellagitannis, flavonoids, anthocyanins), arils (ellagitannis, flavonoids, anthocyanins), seeds (fatty acids, lipids), and membranous walls (mostly ellagitannins). Anthocyanin biosynthesis occurs in parallel in the arils and in the fruit peel. These two tissues are not necessarily correlated in their activity with respect to color production, and often, the two tissues display different colors (Holland et al., 2009; Dafny-Yalin et al., 2010). The same situation could appear in other biochemical pathways responsible for other important metabolites.

High variability was reported for pomegranate fruit that manifests, among other phenomena, considerable differences in size, shape, color, date of ripening, and taste. This external variability is interesting in view of the fact that the only edible species among the *Punica*, which include only two species, is the cultivated pomegranate (*P. granatum* L.). The only other pomegranate known to science is the non-edible species *Punica protopunica*, endemic to Socotra (Holland et al., 2009). The fruit of this species is small and not colorful and no biochemical, genetic, or molecular studies of its fruit were published. This high variability is also reflected in the content of primary and secondary metabolites. Quite substantial work has been devoted in recent years to determining primary metabolites in the pomegranate fruit. These efforts include studies of sugar, organic acids, protein, amino acids, and lipid content and composition. In general, the pomegranate fruit consists of 50% peel, 40% arils, and 10% seeds (per weight). The arils contain 85% water, 10% total sugars, 1.5% metabolites and bioactive compounds such as organic acids, phenolics, and flavonoids (Tezcan et al., 2009). The seeds are a rich source of lipids; pomegranate seed oil comprises 12–20% of total seed weight (Viuda-Martos et al., 2010). It appears that primary and secondary metabolites showed extensive variability due to the fact that the fruit used for the various studies originated from different varieties and highly variable climatic conditions and taken from trees grown under different agro-technical methods. While pomegranate reviews published up until now focused mainly on secondary metabolites (e.g., polyphenols, anthocyanins), there are only few that focused on primary metabolites, despite their great importance to taste attributes and to the nutritional index of the fruit. In this review, we focus on primary metabolites and on secondary metabolites, anthocyanins and hydrolizable tannins, with special attention to the variability of their content and composition. A special effort was aimed at the developmental, genetic, and environmental effects on the content and composition of primary metabolites. Whenever available, primary metabolites in each of the fruit organ, peel, arils, and seeds, were specified.

## Primary Metabolites

### Sugars

**Figure 1:**
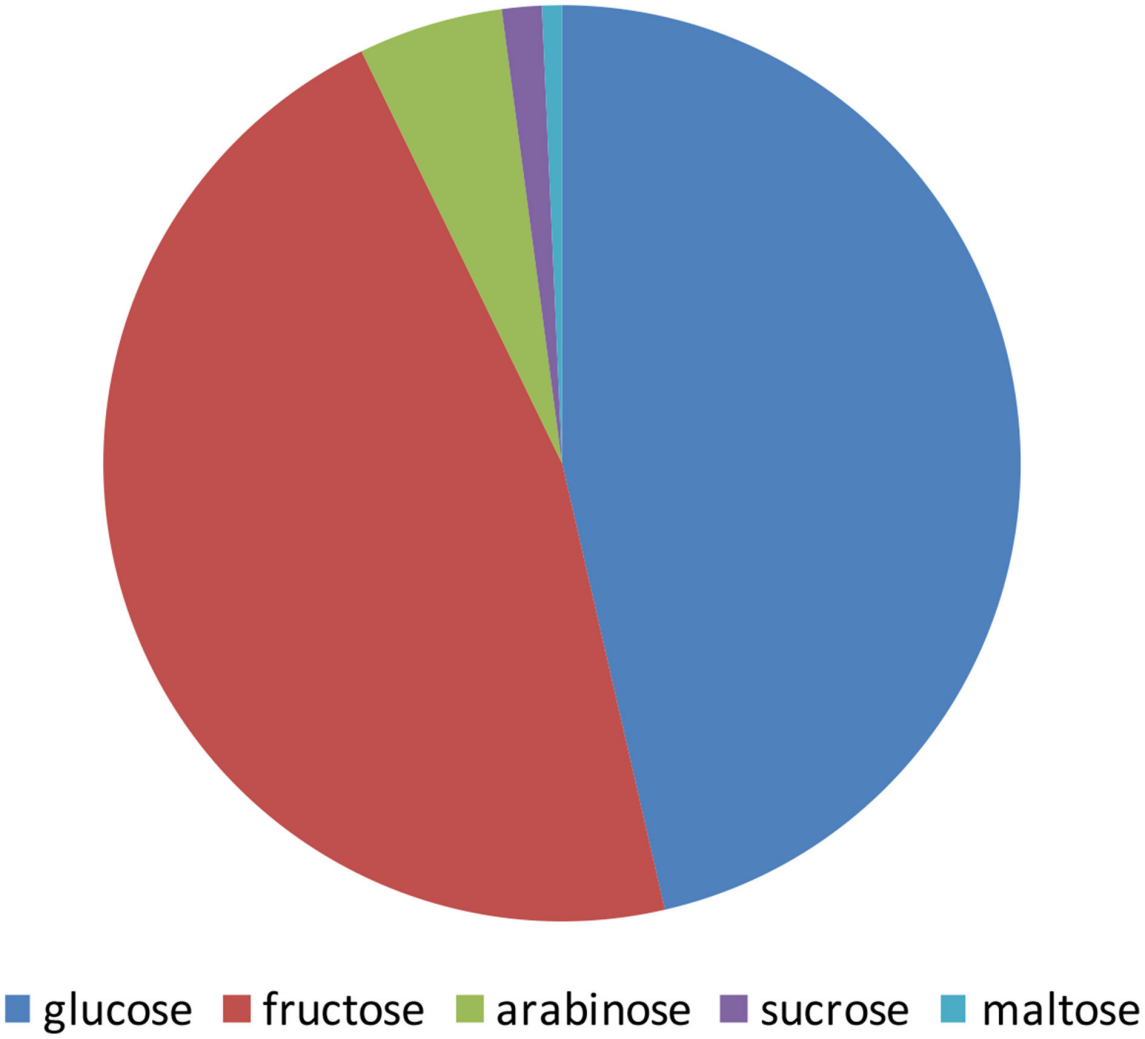
Schematic illustration of the main sugars content in pomegranate juice from different varieties grown in Tunis, Turkey, Spain, and Israel. The values presented are average values of the percentage of each sugar of the total amount of sugars measured by Melgarejo et al. (2000); Tzulker et al. (2007); Hasnaoui et al. (2011); Caliskan and Bayazit (2012).

The pomegranate fruit is a rich source of sugars. The level of the sugars in pomegranate juice is highly correlated with the level of total soluble solids (TSS). Shwartz et al. (2009) and Dafny-Yalin et al. (2010) calculated a value of *R*^2^ = 0.89, *P* < 0.01. The TSS level in the juice ranges from 4.2 to 8.5 g/100 g depending on cultivars, climatic conditions, and cultural techniques (reviewed by Kalaycioglu and Erim (2017); Amir et al. (2018)). Pomegranate juice contains a high amount of polyphenols such as flavonoids, ellgitannins, and the color molecules anthocyanins. A substantial fraction of these molecules are known to be conjugated to sugars, mostly glucose. The taste of arils from various pomegranate varieties is significantly variable, ranging from sour to sweet (Holland et al., 2009; Amir et al., 2018). Sugar content is an important parameter influencing taste, although it is highly influenced by organic acid content as well. Many studies examined the sugars in the pomegranate fruit, mainly in the juice, revealing glucose, and fructose as the main component of the juice sugars (Figure 1). Sugars found in the fruit peel were in some controversy among studies from different countries. It should be noted that those studies were done for different purposes and therefore followed different procedures of extraction and detection that might explain this disagreement. Some of the studies indicated glucose and fructose as the main sugars while others found that xylose and arabinose are the main sugars (Hasnaoui et al., 2014).

#### Differences Among Varieties

##### Composition of sugars in the juice

Arils are a rich source of sugars. Studies obtained from different countries have shown that the composition of sugars among pomegranate varieties might differ. Analyses of the sugars in pomegranate aril juice from 29 worldwide varieties grown in Israel and 19 cultivars from Spain have shown that fructose and glucose were the major sugars found in the arils, while sucrose and maltose were detected in lesser amounts. In some varieties, these two former sugars are the only sugars that were detected (Melgarejo et al., 2000; Dafny-Yalin et al., 2010). In many studies the levels of fructose were similar to those of glucose in pomegranate juices, and both varied in different varieties by a factor of up to two-fold ranging from 4.2 to 8.5 g/100 g (Kalaycioglu and Erim, 2017; Amir et al., 2018). In 76 Turkish varieties glucose levels showed a range of 4.2–8.3 g/100 g juice (Caliskan and Bayazit, 2012). Forty Spanish varieties showed a range of 5.5–7.8 g/100 g (Melgarejo et al., 2000), 29 Israeli varieties showed a range of 4.8–6.6 g/100 g (Tzulker et al., 2007), and 30 Tunisian varieties showed a range of 5.7–8.5 g/100 g (Hasnaoui et al., 2011). In addition to the main fructose and glucose sugars, some other sugars (arabinose, sucrose, and maltose) were also detected in several varieties at a relatively negligible level (Figure 1). Examination of 30 Tunisian varieties that showed low concentrations of arabinose and sucrose (9- and 23-fold lower than glucose, respectively; Hasnaoui et al., 2011). Determination of sugar contents in 6 Spanish varieties has shown that they all contain maltose and sucrose, but possess 45- and 70-fold lower quantities of glucose, respectively (Legua et al., 2012). Sucrose was also found in an about 33-fold lower quantity than glucose in 6 Turkish varieties (Ozgen et al., 2008) and an up to 13-fold lower quantity in 53 out of 76 varieties (Caliskan and Bayazit, 2012). Maltose was only detected in one of 29 Israeli varieties (Dafny-Yalin et al., 2010).

In line with the sugar measurements, the aril juice TSS have shown a relatively narrow range as reported in different publications from different varieties in diverse pomegranate collections (reviewed by Kalaycioglu and Erim (2017); Amir et al. (2018)). Examination of 20 varieties from Iran and Spain, 10 from Morocco, 29 from Israel, and 9 from Italy have shown ranges of 11.4–15.1; 15.1–17.7; 15.2–17.6; 13.7–17.8, and 13.6–18.5%, respectively (Tzulker et al., 2007; Tehranifara et al., 2010; Legua et al., 2012; Ferrara et al., 2014; Alcaraz-Mármola et al., 2017).

##### Composition of sugars in the peel

Several studies specifically measured the level of sugars and TSS in the fruit peel (Dafny-Yalin et al., 2010; Orak et al., 2012; Hasnaoui et al., 2014; Ahmadi Gavlighi et al., 2018). Notably, the major sugars that were detected in the peels differed between the varieties grown in Tunisia, Iran and Israel. In the 12 Tunisian varieties, xylose and arabinose represented more than 60% of the total content, followed by galactose (14%), glucose (~10%), mannose (~5%), rhamnose (~4%), and fucose (~1.5%) (Hasnaoui et al., 2014). Peels from one Iranian variety showed that the main sugar is glucose (44.9–68.1%), followed by galactose (14.6–19.4%), mannose (3.4–18.1%), arabinose (3.1–18.1%), and rhamnose (3.5–6.0%) (Ahmadi Gavlighi et al., 2018). However, in the 29 worldwide varieties grown in Israel, the major sugars were glucose and fructose. The level of glucose varied in the range of 0.9–4.8 g/100 g (5.3-fold), and that of fructose in 0.9–6.6 g/100 g (6.6-fold). The level of fructose was higher than that of glucose in most of the varieties. Maltose was found at an about 50-fold lower concentration than that of glucose and fructose, in a range of 0.8–48.9 mg/100 g, while sucrose was detected in only 6 varieties at relatively low levels (up to 3.1 mg/100 g). Mannitol was also detected in all the varieties, ranging from 10 to 300 mg/100 g (Dafny-Yalin et al., 2010).

As expected from these results, the TSS varied between the different collections. In 12 Tunisian cultivars, it ranged from 16.8 to 19.6 g/100 g (Hasnaoui et al., 2014), which was more than the range of three Indian varieties that show a range of 13.7–14.5 g/100 g (Dzugan et al., 2018). However, these values were much higher than the results reported for 4 Turkish varieties that ranged from 3.8 to 6.4 g/100 g (Orak et al., 2012), and the 29 varieties grown in Israel, which showed a range of 5.2–11.3 g/100 g. In these varieties, the peels had a 2- to 3-fold lower level of TSS compared with the aril juice (Dafny-Yalin et al., 2010).

#### Differences During Fruit Development

Several studies followed the changes in the levels of sugars and TSS in aril juice during fruit development. The results taken from three cultivars (American and Indian) in South Africa (Fawole and Opara, 2013c; Mphahlele et al., 2016), and two cultivars (Israeli and American) from Israel (Shwartz et al., 2009) have shown that the level of TSS rose during the development process in accordance with the levels of glucose and fructose. The increase shown in the two Israeli grown varieties and in “Wonderful” in South Africa was significant (Schwartz et al., 2009; Mphahlele et al., 2016). The presented data indicate that the developmental stage of the pomegranate fruit is associated with sugar accumulation.

#### Climate and Geographic Influence

Analysis of TSS and sugar content in different collections revealed that their values depended on climate and growth conditions. To gain more knowledge on the effect of the environmental conditions on the levels of sugars, 11 varieties from the Israeli collection in the Jezreel Valley (Mediterranean climate) were planted in Israel's southern Arava Valley (hot-dry desert climate). Arils from both habitats were analyzed. The varieties grown in Mediterranean climate showed significantly higher levels of glucose and fructose in the juice than those grown in a hotter habitat (Schwartz et al., 2009). Similar results were also reported from the analyses of the 10 Chinese cultivars that grew in four different habitats (Li et al., 2015). “Wonderful,” which was grown in Israel in two habitats (Ben-Arie et al., 1984), as well as in three habitats in South Africa (Mphahlele et al., 2016), showed that relatively higher temperatures can decrease sugar content, whereas cooler temperatures apparently promoted the increase in glucose and fructose. Thus, temperatures appear to play an important role in the sugar content of pomegranate juice.

#### Genetics

Only one study concerning the genetic control of sugar content in pomegranate was reported. Sugar content expressed as TSS in aril juice was mapped using an F2 population. Two QTLs were detected on linkage group 2 of the genetic map with a LOD score of about 6 and separated by a distance of 20 cM (Harel-Beja et al., 2015).

### Organic Acids

Analyses of the organic acids of pomegranate aril juice have shown that citric acid is generally the predominant organic acid and its content can reach up to 3.76 g/100 g in the juice. In addition, it contains significantly lower levels of malic, oxalic, succinic, tartaric, and ascorbic acids (Table 1). In the fruit peel, citric acid is the predominant organic acid and its content can reach up to 1.68 g/100 g. Smaller amounts of malic, succinic, and oxalic acid were also detected in peels.

**Table 1:** The levels of organic acids (g/100 g juice) and total titratable acidity (%) in aril juices of different varieties from different collections grown in different countries.

**Variety no**.	**Growing country**	**Citric acid**	**Malic acid**	**Oxalic acid**	**Succinic acid**	**Tartaric acid**	**Ascorbic acid**	**Titratable acidity (%)**	**References**
10	Morocco	0.00–3.20	0.30–1.50	nd	0.03–0.37	nd	nd	2.4–37.5 (15.6)	Legua et al., 2012
15	Spain	0.06–1.85	0.09–0.14	nd	nd	0.02–0.04	nd	1.9–14.3 (7.5)	Mena et al., 2011
40	Spain	0.08–0.25	0.08–0.21	0.01–0.07	nd	0.00–0.01	nd	2.1–12.4 (5.9)	Melgarejo et al., 2000
29	Israel	0.20–2.00	0.02–0.60	0.00–0.42	0.00–0.26	nd	0.06–0.12	0.2–3 (15)	Dafny-Yalin et al., 2010
30	Tunisia	0.04–3.14	0.72–2.04	0.03–0.65	0.14–0.89	0.00–0.18	nd	0.2–3.4 (16.7)	Hasnaoui et al., 2011
20	Spain	0.04–1.90	0.35–1.20	nd	nd	nd	nd	1.4–19.2 (13.7)	Alcaraz-Mármola et al., 2017
13	Turkey	0.03–0.90	0.06–0.69	0.00–0.67	0.00–0.15	0.03–0.28	nd	4.6–17.3 (3.8)	Poyrazolua et al., 2002
7	Turkey	0.39–1.31	0.03–0.24	nd	nd	nd	nd	nd	Tezcan et al., 2009
6	Turkey	0.20–3.20	0.09–0.15	nd	nd	nd	0.01–0.06	0.5–3.8 (7.6)	Ozgen et al., 2008
25	Iran	0.00–3.76	0.02–0.37	0.01–0.06	0.00–0.13	0.03–0.11	0.00–0.01	nd	Aarabi et al., 2008

*nd, not detected; different units used in different studies were converted (1 g juice is equivalent to 1 ml juice); Numbers in bracket are the fold change between the values*.

#### Differences Among Varieties

Differences in organic acid composition in aril juice as well as in peels were detected among pomegranate varieties.

##### Composition of organic acids in the juice

The citric acid's level varies significantly between the different varieties. A range of 0.4–31.4 g/L, 78-fold difference in citric acid content was found among 12 Tunisian varieties (Hasnaoui et al., 2011); 0.08–0.25 g/100 g, 30.7-fold variance among 40 Spanish varieties (Melgarejo et al., 2000); 0.33–8.96 g/L, 27-fold among 13 Turkish varieties (Poyrazolua et al., 2002); and 0.2–2.0 g/100g, 22-fold among 29 Israeli varieties (Dafny-Yalin et al., 2010) (Table 1). In addition to citric acid, other organic acids were detected in the juice of pomegranates. These include malic, succinic, and oxalic acids (Table 1). However, the levels of these organic acids were relatively low compared to those of the citric acid. In addition, traces of ascorbic, acetic, tartaric, quinic, fumaric, maleic, and lactic acids were detected in some accessions (Melgarejo et al., 2000; Poyrazolua et al., 2002; Aarabi et al., 2008). This suggests that citric acid is a major component of acidic taste in pomegranate fruits. Indeed, the level of citric acid shows a strong and positive correlation with total titratable acidity (*R*^2^ = 0.91, *P* < 0.01) as measured in several collections (e.g., Poyrazolua et al., 2002; Tzulker et al., 2007; Hasnaoui et al., 2011). This value significantly changes between the different varieties. The values were about 15-fold in a study of 29 varieties in Israel (Dafny-Yalin et al., 2010), 10 varieties from Morocco and 40 from Tunisia (Hasnaoui et al., 2011; Legua et al., 2012).

Since the level of sugars does not change much and that of acidity differs considerably among the varieties, acidity level is considered to be the main factor that determines the variability of taste in arils (Ben-Arie et al., 1984; Lobit et al., 2003). The ratio of TSS to total acidity values ranged significantly from 6.1 to 64.6 among 29 Israeli-grown varieties when the low values stand for the sour pomegranates and the high for the sweet fruits (Dafny-Yalin et al., 2010). Measurements of 40 Spanish varieties revealed that in sour taste fruits, this ratio ranged from 32 to 96, and in fruits with a sour-sweet taste the values were 17–28 (Gil et al., 1995b). In sweet and sour varieties in Italy the TSS to total acidity ratio ranged from 6.6 to 35.2 (Ferrara et al., 2011).

##### Composition of organic acids in the peel

As in the arils, the major organic acid in the peel is citric acid, but its level was about 3- to 5-fold lower compared to its level in the arils. The citric acid levels vary from 11 to 390 mg/100 g (13-fold) among the 29 cultivars (Dafny-Yalin et al., 2010). Besides citric acid, malic acid (1.5–32 mg/100 g) and succinic acid (2.5–14 mg/100 g) were also detected in the peel. Eight out of 12 cultivars containing oxalic acid in their arils also contained oxalic acid in their peel (6–31 mg/100 g) (Dafny-Yalin et al., 2010). Citric acid was also the dominant organic acid (507–1,678 mg/100 g) in the peels of six cultivars grown in Georgia, followed by malic (93–116 mg/100 g) and succinic acids (13–16.5 mg/100 g), while oxalic acid was found at a lower level (7.4–9.3 mg/100 g) (Pande and Akoh, 2009). Total acidity values in peels ranged in four Turkish cultivars from 1.48 to 3.66% (Orak et al., 2012), from 0.27 to 1.23% in 29 Israeli varieties (Dafny-Yalin et al., 2010), from 0.97 to 1.39% in five Turkish cultivars (Gözlekçi et al., 2011), and from 0.36 to 0.40% in three Indian cultivars (Dzugan et al., 2018). The level of organic acids in pomegranate peel appears to be highly variable. The reasons for this could be manifold and depend on genetic background, methods of extraction and fruit ripening stage at harvest time. It appears that the developmental stage of the pomegranate fruit in sour cultivars is associated with reduction in total acidity.

#### Differences During Fruit Development

Several studies followed the levels of organic acids and titratable acidity in aril juice during fruit development. Indian and Tunisian cultivars grown in India (“Ganesh” and “Taifi”) (Kulkarni and Aradhya, 2005), and Israeli and American cultivars grown in Israel (“Rosh Hapered” and “Wonderful”) (Shwartz et al., 2009) showed that total acidity decreased during fruit maturation. Respectively, the TSS to total acidity ratio and the pH increased across all the tested cultivars.

During 10 weeks of the development and ripening of two cultivars grown in Israel (“Wonderful” and “Rosh Hapered”), the level of citric acid as well as that of total acidity in “Wonderful” decreased significantly. The content of citric acid in the sweet “Rosh Hapered” was the lowest compared to malic, succinic and oxalic acids, and was not significantly correlated with total acidity in this cultivar. The levels of malic and ascorbic acids increased in both cultivars during fruit development (Shwartz et al., 2009). Unlike the results obtained from two different studies done in Israel with “Wonderful” (Ben-Arie et al., 1984; Shwartz et al., 2009), in South Africa, citric acid and succinic acid increased during the development of “Wonderful” while malic acid did not significantly change (Mphahlele et al., 2016). This increase in citric acid was observed despite the fact that total acidity decreased from 2.1 to 1.1 g/100 mL. Analysis of the levels of ascorbic acid (Vitamin C) reveals that its level decreased significantly during the development of Tunisian and Indian cultivars (Al-Maiman and Ahmad, 2002; Kulkarni and Aradhya, 2005). However, in the two cultivars that were grown in Israel, the content of ascorbic acid increased (Shwartz et al., 2009) during development. The differences in the content of citric acid in “Wonderful” between South Africa and other countries are intriguing in view of the fact that citric acid is a major organic acid that contributes to acidity and that total acidity decreased in all studies, including the one from South Africa.

#### Climate and Geographic Influence

Total titratable acidity values were shown to be affected by climate and growth conditions. Arils from 11 varieties grown in the Jezreel Valley (Mediterranean climate) and in the Southern Arava Valley (hot-dry desert climate) were analyzed to study the effect of environmental and climatic conditions on the arils' acid content. The cultivars grown in Mediterranean climate had higher acidity levels compared to the acidity levels found in desert climate. This was in accordance with the higher contents of citric and malic acids, the two main organic acids in the arils (Schwartz et al., 2009).

Generally sour cultivars are mostly grown in northern cold regions, while sweet cultivars with low acidity values are mostly found in regions having hot dry conditions. In Southern Spain and North Africa most of the commercialized cultivars have a sweet taste (Al-Kahtani, 1992), while in North Turkey and Russia sour cultivars are commercialized (Gabbasova and Abdurazakova, 1969; Mayuoni-Kirshinbaum and Porat, 2014; Alcaraz-Mármola et al., 2017). In northern regions such as Russia, Macedonia, Georgia, and Turkey, the total acidity ranged from 0.5 to 2.3% (Gabbasova and Abdurazakova, 1969), 0.6 to 2.2% (Pande and Akoh, 2009), 0.4 to 2.8% (Veres, 1977), and 1.7 to 4.6% (Poyrazolua et al., 2002), respectively. However, in hot climates such as in India, Egypt, and Saudi Arabia, total acidity values dropped to 0.12–0.13% (Al-Maiman and Ahmad, 2002), 0.03–0.10%, and 0.02–0.14%, respectively (Al-Kahtani, 1992).

### Amino Acids

Amino acids are organic compounds that among other functions have an important role in protein biosynthesis and secondary metabolite syntheses. In addition to their role as building blocks of proteins, amino acids function as precursors or intermediates in biosynthetic pathways such as production of color molecules and volatiles in fruits, energy release through degradation, signaling processes in plant metabolism regulation and plant stress response (Creighton, 1993; Tatjana et al., 2015; Li et al., 2017). Plants are a nutritional source for these elements and hence the importance of amino acid availability in fruits. There are very limited data and only a handful of research publications concerning amino acid in the pomegranate fruit.

#### Composition of Amino Acids in the Juice

**Figure 2:**
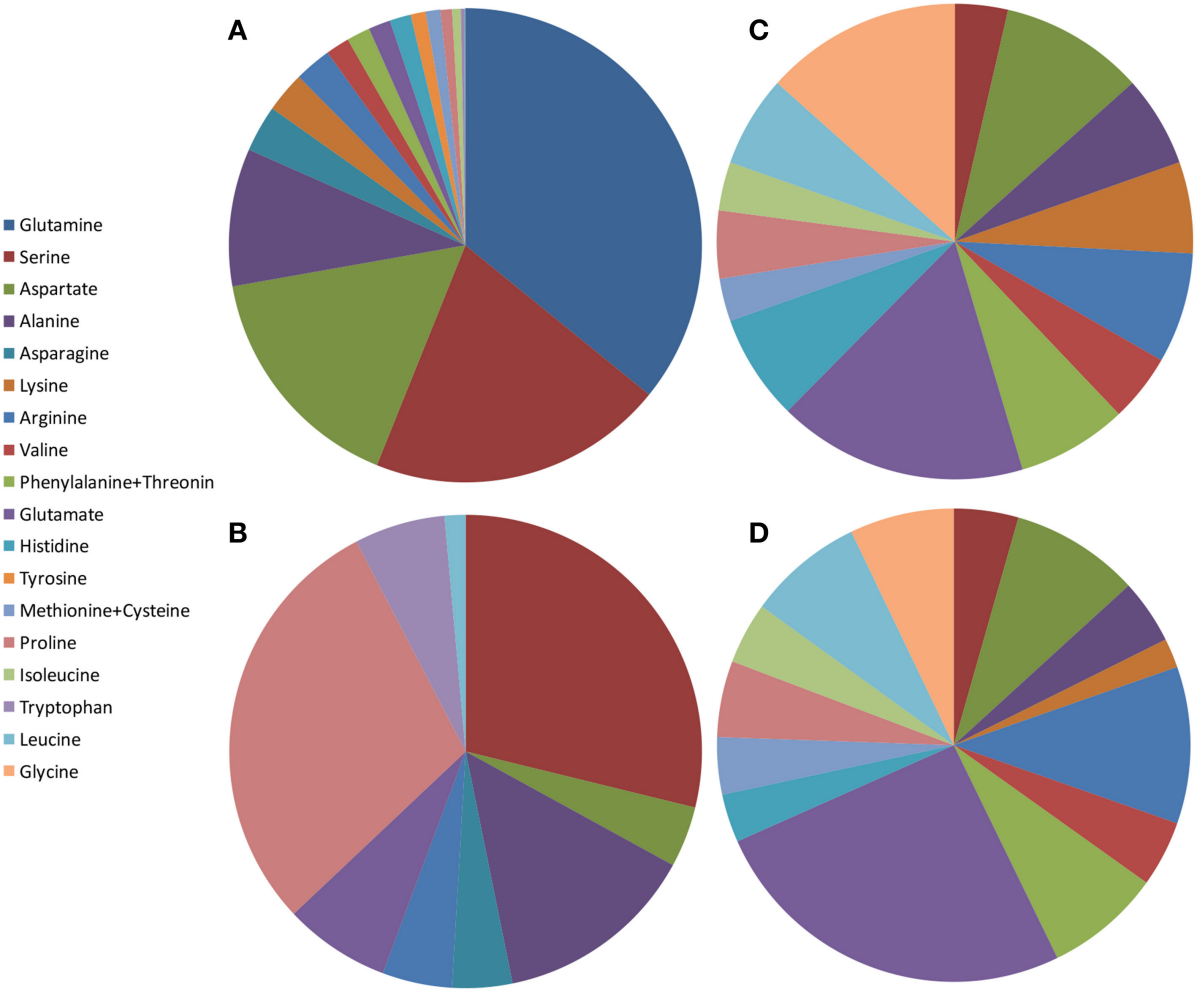
Schematic illustration of amino acid content in pomegranate fruit tissues from different varieties and countries. The values presented are average values of the percentage of each amino acid of the total amount of amino acids measured: **(A)** juice from China by Li et al. (2017); **(B)** juice from Turkey by Tezcan et al. (2013); **(C)** peel powder from Egypt by Rowayshed et al. (2013); **(D)** seed powder from Tunisia and Egypt by Elfalleh et al. (2011) and Rowayshed et al. (2013).

There are just two studies involving amino acid profile in pomegranate juice and they are incomparable (Figure 2). In this context, it should be pointed out that different detection methods might result in different amino acid compositions. Thus, one cannot conclude in general the composition of amino acids in pomegranate juices excluding the fact that serine is found at high percentages in all juices studied.

Li et al. (2017) studied amino acids in juices of separated arils of six Chinese cultivars from two regions. Glutamine, serine, aspartate, and alanine are the most abundant amino acids in the juice, while glycine was not detected. All the essential amino acids exist but in smaller portions (Figure 2A). Tezcan et al. (2013) analyzed fresh squeezed juice from three pomegranate fruits and three commercial pomegranate juices obtained from local markets (claimed to be 100% pomegranate). The authors did not specify if the juice was squeezed from separated arils or from the intact arils. In addition to the L-amino acids that were identified, D-proline was detected in all the juices and D-leucine in one of the commercial juices. L-serine, L-proline, and L-alanine are the most abundant amino acids in these pomegranates juices, while D-leucine was not detected (Figure 2B).

#### Composition of Amino Acids in the Peel

Rowayshed et al. (2013) studied the peel powder of local Egyptian fruit obtained from the market. They found that glutamine (0.52 g/100 g), glycine (0.41 g/100 g), and aspartate (0.3 g/100 g) are the most abundant amino acids in the pomegranate peel powder studied. Tryptophan was not detected but all the other essential amino acids exist (Figure 2C). Asparagine, glutamine, and tyrosine were not measured in this study. The peel powder studied contained an exceptionally higher content of lysine, isoleucine, methionine, and cysteine than the reference protein pattern of FAO/WHO (Rowayshed et al., 2013).

#### Composition of Amino Acids in the Seeds

There are only two studies involving amino acid profiling in pomegranate seeds (Elfalleh et al., 2011; Rowayshed et al., 2013). Elfalleh et al. (2011) studied amino acids in seeds of two Tunisian commercial pomegranate cultivars and Rowayshed et al. (2013) studied local Egyptian varieties. Both revealed high proportions of glutamate (3.5 g/100 g), arginine (1.9 and 1.47 g/100 g respectively), and aspartate (1.9 and 1.21 g/100 g, respectively) in dry seeds. Figure 2D shows the average results of these studies as percent of each amino acid of the total amino acids measured. Notably, the amino acid composition in seed powder was very similar to those found in peels of local Egyptian fruit (Rowayshed et al., 2013).

The two groups (Elfalleh et al., 2011; Rowayshed et al., 2013) found that essential amino acid content is much higher than the requirement of FAO/WHO for adults. Since these essential amino acids are usually deficient in most foods, the authors suggested that these tissues can serve as food supplements.

#### Differences Among Varieties

A study on juices of six pomegranate cultivars grown in two regions in China revealed that the genotype of the pomegranate had a significant effect on the amino acid profile and content (Li et al., 2017). Clustering analysis relying on amino acid content showed segregation of amino acids in these pomegranate cultivar juices. This difference was mainly attributed to the differences in the content of cysteine, ornithine, aspartate, serine, methionine, and leucine. Analysis of proline in three Turkish cultivars from three regions in two successive years show that proline varied among pomegranate cultivars (Halilova and Yildiz, 2009) although the data published show insignificant difference between the cultivars (i.e., 65 ± 31, 60 ± 30, and 59 ± 33 mg/L). Elfalleh et al. (2011) studied amino acids in seeds of two Tunisian commercial pomegranate cultivars. No significant differences were found between “Jebali” and “Gabsi” cultivars but significant differences (*p* < 0.05) were observed in the levels of glycine, cysteine, methionine, histidine, arginine, and proline (Elfalleh et al., 2011).

#### Differences During Fruit Development

No information is available regarding amino acid content changes through fruit development except for the information given by Nuncio-Jáuregui et al. (2014) for proline. This group evaluated the effects of fruit maturation stage on pomegranate juices' chemical structure. A positive relationship between fruit maturation stage and proline content was reported in different cultivars, showing that proline increased from 32 to 84 mg/L in “Mollar de Elche” juices (Nuncio-Jáuregui et al., 2014).

#### Climate and Geographic Influence

Li et al. (2017) studied six pomegranate cultivars from Shandong (near the ocean) and Xinjiang (Eurasian continental climate) regions in China. The pomegranate juices from separated arils from Shandong had higher levels of total amino acids and of essential amino acids than those from Xinjiang. The total glutamate-, aspartate-, pyruvate-, and serine-related amino acids were higher in the Shandong juices, while the total aromatic amino acid contents were higher in the Xinjiang juices.

Halilova and Yildiz (2009) quantified proline content in freshly pressed juice of three Turkish cultivars from three regions in 2 successive years. The authors found that the average proline content was 30 mg/L in the first year and 93 mg/L in the second, which was drier and hotter than the first year. The authors claim that this 3-fold increase in the second year indicates that climatic change affects proline accumulation (Halilova and Yildiz, 2009).

According to the researches, it can be concluded that amino acid content in pomegranate fruits is influenced by environmental conditions, particularly temperature and water availability.

### Proteins

The data on proteins in pomegranate fruits are limited and mainly concern total protein content in various tissues. Most of the studies do not report specific protein functions with the exception of storage proteins in the seeds and lipid transfer proteins in the arils. In general, the percent of total proteins in pomegranate juice is usually low, from <1.0 to 1.1%, which is quite a narrow range. Diversely, the percent of total proteins in pomegranate seeds varies from 4.1 to 16.9%, which is quite a wide range. In this tissue, the presence of the storage proteins, globulins, albumins, glutelins, and prolamins, is prominent and the first two are the major proteins found in most of the studies.

#### Differences Among Varieties

##### Composition of proteins in aril flesh and juice

Elfalleh et al. (2011) studied proteins in the juice and pulp of two Tunisian commercial pomegranate cultivars. Protein content in the juice was 7.95 ± 0.89 g/L and differed significantly between the two cultivars (Elfalleh et al., 2011). Elfalleh et al. (2009) studied fresh pomegranate juices (from arils only) and dry pulps of fully mature fruits from six local Tunisian ecotypes. The juice protein content was about 6.67 ± 2.26 g/L and the dry pulp protein content was 22.9%. The juice protein contents were significantly different, varying from 9.93 ± 1.90 g/L in “Chetoui” to 4.13 ± 1.20 g/L in “Gabsi 2.” The authors state that pomegranate is highly proteinic (~0.66%) compared to red wine (0.04%) and raw apple juice (0.27%) (Elfalleh et al., 2009). Al-Maiman and Ahmad (2002) studied total protein amounts in the aril juice of Saudi Arabian pomegranate “Taifi.” Fully-ripe fruits contained 1.05% protein in the juice (Al-Maiman and Ahmad, 2002). Kulkarni and Aradhya (2005) report total protein conteny of about 85 mg/100 g in the juice of ripe Indian “Ganesh” (100 days after fruit set).

##### Composition of proteins in the seeds

El-Nemr et al. (1990) determined crude protein in fully ripened Egyptian pomegranate fruits obtained from the local market. The analysis discovered that seeds, but not juice, contain protein and that 13.2% of the constituents measured in the dry seeds were unidentified proteins (El-Nemr et al., 1990). Al-Maiman and Ahmad (2002) studied total protein amounts in seeds of Saudi Arabian pomegranate “Taifi.” Fully-ripe fruits contained 4.06% of protein in the seeds (Al-Maiman and Ahmad, 2002). Elfalleh et al. (2011) studied proteins in the seeds of two Tunisian commercial pomegranate cultivars. Seed storage protein content was 167.8 ± 8.9 mg/g dry weight, which constitutes 16.9% of the seeds' dry weight. Globulins (62.4 mg/g) and albumins (54.1 mg/g) are its major fractions, followed by glutelins (33.2 mg/g) and prolamins (18.1 mg/g). No significant difference in the content of albumins and glutelins was found between the cultivars (Elfalleh et al., 2011). Elfalleh et al. (2010) studied storage proteins in seeds of mature pomegranate fruits of eight different Tunisian cultivars from five Tunisian regions. The authors reported that the seeds contain 16.8% proteins (per dry weight). The pomegranate seeds accumulated mainly globulins (43%) and albumins (32%). Glutelins constituted 16% and prolamins only 9% of the proteins found. Significant differences between the cultivars in the total amount of storage proteins, ranging from 15.4% in “Beldi” to 20.1% in “Rafrafi” was found, as well as differences for each fraction's content (Elfalleh et al., 2010). Zang (2011) determined protein content in pomegranate seeds oil originating from Xinjiang. The average content of crude protein in pomegranate seeds was 14.3%, in which glutelins and residual protein constituted more than 80% of total protein content, and the contents of globulins, albumins, and prolamins were lower (Zang, 2011).

In summary, it appears that there are differences in total protein content between pomegranate varieties. There is also variability in the content of the different storage proteins in the seeds. This variation may be connected to their genetic background but also to different analysis methods or environmental conditions.

#### Differences During Fruit Development

Al-Maiman and Ahmad (2002) studied total protein amounts in aril juice and seeds of Saudi Arabian pomegranate “Taifi” and compared unripe, half-ripe, and fully-ripe fruits. Protein concentration in seeds was found to be about four times higher than in the juice (an average of 4.06 vs. 1.05%). No significant changes were observed in protein concentration during fruit development in the seeds. The juice of unripe fruits contained significantly less proteins than the quantities in half-ripe and fully-ripe fruits (Al-Maiman and Ahmad, 2002). Kulkarni and Aradhya (2005) reported total protein content in squeezed separated arils of the Indian “Ganesh” at seven fruit developmental stages (20, 40, 60, 80, 100, 120, and 140 days from fruit set). The study revealed significant changes in total protein content in the juice during fruit development. The highest total protein (209 mg/100 g) occurred 20 days after fruit set with a rapid decrease (66.9%) toward 80 days after fruit set. An increase (58.7%) occurred from 80 to 120 days and a significant slight decrease (6.3%) in total protein content occurred after 120 days (Kulkarni and Aradhya, 2005).

These two studies indicated that total protein content in the juice changes during pomegranate fruit development, but this does not happen in the seeds.

### Lipids

Lipids are a group of small hydrophobic molecules that include fatty acids, waxes, sterols, fat-soluble vitamins, phospholipids, mono-, di-, and triglycerides. Primary and secondary lipids have diverse functions in living organisms, including energy storage, cell signaling, nutrition (fats and vitamins), hormones, transport, and structural components of cell membranes. The most lipid-rich fraction in pomegranates is the seeds, which contribute 10% to fruit weight. Generally, seed oil constitutes 6–20% of seed weight and contains a large quantity of lipids (Viuda-Martos et al., 2010; Ferrara et al., 2011). The chain length of the lipids is divided to three classes: Medium- (C6–C12), long- (C14–C20), and very long (C22 and C24). The total lipid (the term refers to primary and secondary lipids) percentage in seeds varies from 4.4 to 27.2% (El-Nemr et al., 1990; Pande and Akoh, 2009; Jing et al., 2012; Ferrara et al., 2014; Verardo et al., 2014; Fernandes et al., 2015a). The list of lipids found in pomegranate fruit tissues is presented in Table 2.

**Table 2:** Lipids including fatty acids, sterols, and triterpens identified in pomegranate fruit peel, aril juice, and seed tissues; (+) reported presence; (–) presence not yet reported.

**Lipid group**	**Lipid molecule**	**Fruit peel**	**Aril juice**	**Seed**
Fatty acid	Arachidic acid	+^a^	+^a^	+
	Behenic acid	–	–	+
	Capric acid	–	+	–
	Caproic acid	–	+	–
	Caprylic acid	–	+	+
	Catalpic acid	–	–	+
	Docosadienoic acid	–	–	+
	Eicosenoic acid	–	–	+
	Eicosapentaenoic acid	–	–	+
	α-Eleostearic acid	–	–	+
	β-Eleostearic acid	–	–	+
	Erucic acid	–	–	+
	Gadoleic acid	–	–	+
	Gondoic acid	–	–	+
	Lauric acid	–	–	+
	Lignoceric acid	–	–	+
	Linoleic acid	+^a^	+^a^	+
	Linolelaidic acid	–	–	+
	α-Linolenic acid	+^a^	+^a^	+
	γ-Linolenic acid	+^a^	+^a^	+
	Margaric acid	–	–	+
	Myristic acid	+^a^	+^a^	+
	Myristoleic acid	–	–	+
	Nervonic acid	+^a^	+^a^	+
	Oleic acid	+^a^	+^a^	+
	Palmitic acid	+^a^	+^a^	+
	Palmitoleic acid	+^a^	+^a^	+
	Pentadecylic acid	–	–	+
	Punicic acid	+^a^	+^a^	+
	Stearic acid	+^a^	+^a^	+
	cis-Vaccenic acid	–	–	+
	Triacylglycerols, 3-O-octadec-2-enoic acid	–	–	+
	Tricosylic acid	–	–	+
	9Z, 11E, 13Z-Octadecatrienoic acid	+	–	+
	8Z, 11Z, 13E-Octadecatrienoic acid	+	–	+
Sterol	Campesterol	–	–	+
	Cholesterol	–	–	+
	Citrostadienol	–	–	+
	Daucosterol	–	–	+
	Estradiol	–	–	+
	Estrone	–	–	+
	Estriol	–	–	+
	β –Sitosterol	–	–	+
	β-Sitosterol laurate	+	–	–
	β-Sitosterol myristate	+	–	–
	Stigmasterol	–	–	+
	Testosterone	–	–	+
	Δ5-Avenasterol	–	–	+
Triterpene	Asiatic acid	–	–	+
	Betulinic acid	–	–	+
	Cycloartnol	–	–	+
	Punicanolic acid	+	–	–
	Squalene	–	–	+
	Ursolic acid	–	–	+
Glycosphingolipid	Glycosphingolipid N-palmitoyl cerebroside	–	–	+
	N-Palmitoyl cerebroside	–	–	+
Glycerolipid	1-*O*-Isopentyl-3-*O*-octadec-2-enoyl glycerol	+	–	+
	1-*O*-Octadecatrienoyl glycerol	–	–	+
	Di-*O*-Punicyl-*O*-octadeca-trienylglycerol	–	–	+
	Tri-*O*-Punicylglycerol	–	–	+
Phospholipid	Phosphatidylethanolamine	–	–	+
	Phosphatidylcholine	+^a^	+^a^	+
Tocopherol	α-Tocopherol	–	–	+
	γ-Tocopherol	–	–	+

a *The molecule was detected in a mix of juice and peels*.

**Figure 3:**
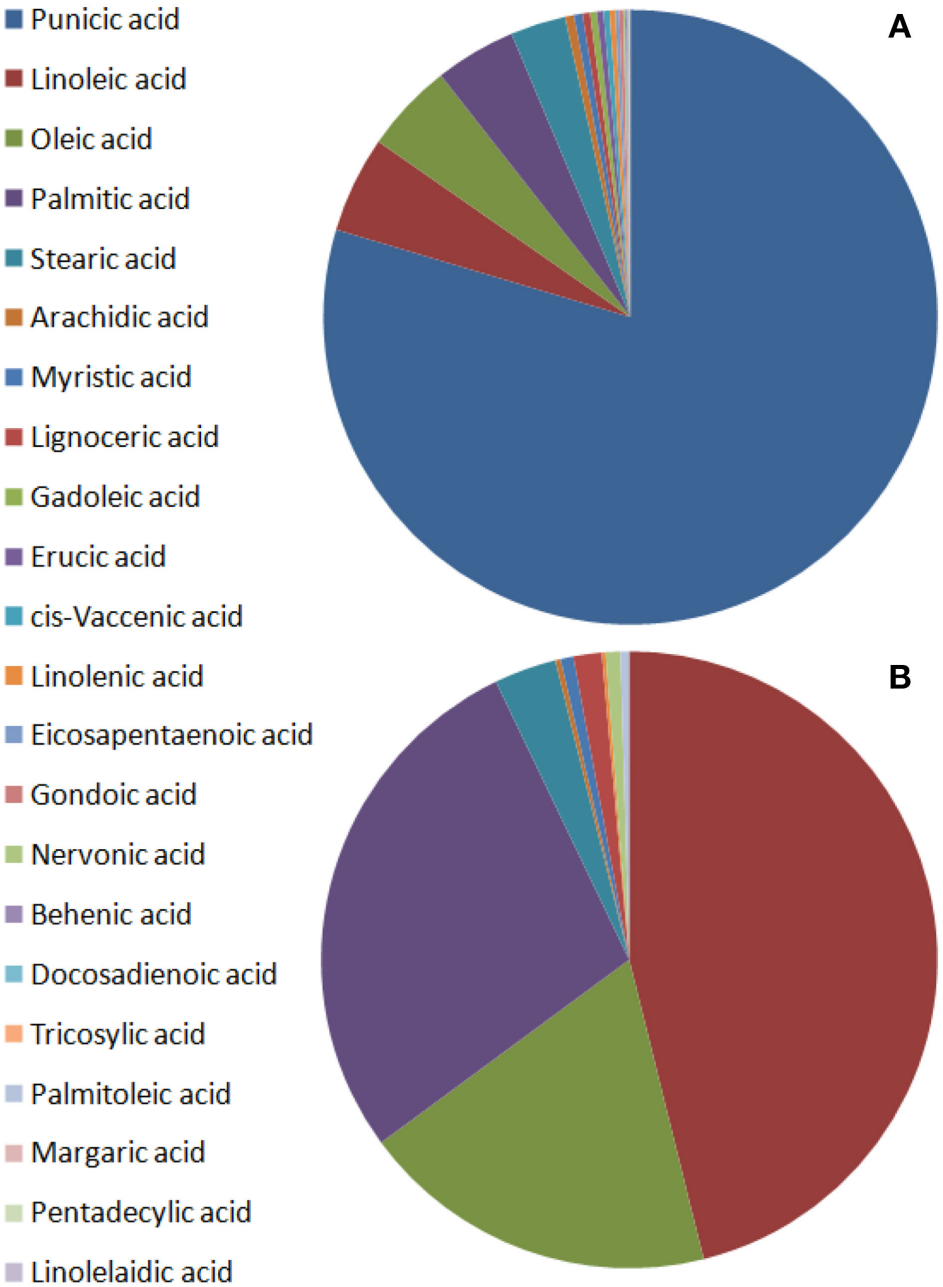
Schematic illustration of fatty acid content in pomegranate fruit tissues from varieties of different origin. The values presented are average values of the percentage of each fatty acid of the total fatty acids measured. **(A)** Seed oils by Pande and Akoh (2009), Ferrara et al. (2014), and Verardo et al. (2014); **(B)** non-seed fruit tissues by Pande and Akoh (2009).

Punicic acid is the most abundant fatty acid in seed oil, constituting over 60% of the fatty acids, mostly followed by oleic acid, linoleic acid, and palmitic acid in a variable order (Figure 3A, Pande and Akoh, 2009; Jing et al., 2012; Ferrara et al., 2014; Verardo et al., 2014; Wu and Tian, 2017). Triterpenoids and phytosterols have been found in pomegranate seed and fruit peel (Seeram et al., 2006b; Verardo et al., 2014; Wu and Tian, 2017). The major phytosterol detected in seed oil is sitosterol (Kaufman and Wiesman, 2007; Pande and Akoh, 2009; Verardo et al., 2014). Most of the lipids were identified in the seeds, small amounts of lipids were also detected in aril juice and fruit peel. It should be mentioned that the presence of some of the lipids such as human steroid hormones is disputed (Choi et al., 2006).

#### Differences Among Varieties

##### Composition of lipids in the seed oil

Verardo et al. (2014) studied the lipid composition (fatty acids, sterols, tocopherols, and phospholipids) of pomegranate seed oil from 17 varieties: (4 Israeli, 3 Spanish, 1 Turkish, 1 Iranian, 2 Tunisian, 6 Italian). The total lipid content (primary and secondary) of the pomegranate seeds varied from 7.6 to 16.2%. The oil consisted of 65–80% conjugated fatty acids of which punicic acid constituted 74–85%. Other major fatty acids were oleic, linoleic and palmitic acid. Polyunsaturated fatty acids constituted 87.2% of the total seed oil, while monounsaturated fatty acids constituted 7.1% and saturated fatty acids constituted 5.7% of the total amount of fatty acids in the seed oil. Total sterol content varied between 7.5 and 16.4 mg/g of oil. The major phytosterols detected were campesterol, stigmasterol, sitosterol, Δ5-avenasterol, and citrostadienol. Sitosterol constituted 65–74% of the total sterols. Triterpene compounds cycloartenol and squalene constituted 0.8–2.4 mg/g oil (45.1%) and 0.7–3.2 mg/g (42.5%), respectively. Phospholipids were 0.4–2.3% of the total lipids and phosphatidylethanolamine was the main compound, constituting 56–86% of total phospholipids. In addition, total tocopherol content ranged between 678.3 and 2627.4 μg/g of oil, and γ-tocopherol, was 91% of the total tocopherols. There were significant differences between the varieties in fatty acids, sterols, phospholipids, and tocopherols. Differences were also found between variants of the same variety. Such are for example differences that were found between the landraces “Wonderful” and “Wonderful 1” in the content of oleic acid (17.34 and 32.07%, respectively), and squelene (0.91 and 3.18 mg/g oil, respectively) (Verardo et al., 2014).

Jing et al. (2012) studied the lipid composition of extracted seed oil from four Chinese cultivars from Shanxi. Oil content in the seeds ranged from 114.2 to 147.9 mg/g and was significantly different between cultivars. Seed oil was rich in polyunsaturated fatty acids (87–92% of fatty acids), which were significantly different in the four cultivars. Punicic acid was the dominant fatty acid (73.5–78.8 g/100 g fatty acids). Total tocopherols ranged from 2,188 to 4,947 μg/g. The four cultivars were significantly different in their lipid content (Jing et al., 2012). Another study in China examined the content and composition of fatty acid in the seed oil of pomegranates from Xinjiang. The oil content was 18.2%, and unsaturated fatty acids were more than 70% of total fatty acid (Zang, 2011).

Ferrara et al. (2014) studied the oil content and fatty acid composition of 13 sweet and sour pomegranate genotypes from Puglia region in Southeastern Italy, of which 3 were of Israeli origin. The oil extracted from the dried seeds and the content of total lipids were significantly variable among these genotypes, ranging from 10.7 to 26.8% in sweet genotypes and from 4.9 to 17.4% in sour genotypes. Sixteen fatty acids were identified in this study, among which punicic acid was the major fatty acid in all genotypes. Punicic acid content exceeded 74.9%, followed by palmitic, linoleic, stearic, and oleic acids. There was low variability of fatty acid composition between the genotypes. Unsaturated fatty acids in the seed oils constituted between 86.7 and 91.2% with saturated/unsaturated ratios ranging between 0.10 and 0.15 (Ferrara et al., 2014).

Fadavi et al. (2006) determined the fatty acid composition of seed oil from 25 Iranian pomegranate varieties. Oil was extracted from dry seeds of commercially ripe fresh fruits from Markazi and Yazd provinces in Iran. Oil content ranged from 6.6 to 19.3% (W/W). The pre-dominant fatty acid was linolenic acid–31.8–86.6%—followed by linoleic acid, oleic acid, stearic acid, and palmitic acid (Fadavi et al., 2006). Worth noticing is the fact that punicic acid was not detected in this study. This fatty acid was found to be the predominant acid in pomegranates in many other studies but was not detected in these varieties. Since punicic and linolenic acids are isomers, this might be a result of different classification or misinterpretation of the molecule's nature. Differences between the varieties were significant. Moreover, differences were found in the lipid contents of sweet, sour, and sour sweet varieties while the lowest was in sweet varieties and the highest in sour sweet.

The profile of fatty acids and phytosterols in pomegranate seed oil from four varieties grown in Israel was determined. Results showed linolenic acid to be the predominant fatty acid (64–83%). The linolenic acid fraction was composed of four different chromatographically separate peaks that are assumed to be attributed to different isomers of conjugated linolenic acid, and punicic acid was the major isomer. Phytosterols were found at quite a high concentration (4,089–6,205 mg/kg) with a wide variety of components, and the major phytosterols were β-sitosterol, campesterol, and stigmasterol (Kaufman and Wiesman, 2007). Fatty acid and tocopherol composition in the seed oil of nine worldwide pomegranate varieties that were grown in Spain was analyzed resulting in 4.4–12.0% oil content. Over 86% of the oil were unsaturated fatty acids, mainly punicic acid, ranging between 77.3 and 83.6% of total fatty acids. Total tocopherols ranged from 174.5 to 627.3 mg/100 g oil, mainly γ-tocopherol. There were significant differences between the cultivars (Fernandes et al., 2015a).

Lipids were also studied in local varieties from some other countries. Fatty acid content was studied in seeds of fully ripened local market Egyptian pomegranate fruits. Total lipids were 27.2% with saturated fatty acids being 83.6% of the total fatty acids. The predominant acid was caprylic acid (36.3%), followed by stearic acid, oleic and linoleic acids out of 11 fatty acids that were identified (El-Nemr et al., 1990). Punicic acid or linolenic acid were not detected. The oil content and fatty acid composition of the seed oil of seven Spanish sweet pomegranate varieties was 6.3–12.2%, of which 73.4–95.8% were unsaturated fatty acids. The predominant fatty acid was linolenic acid (43.4–88.2%), followed by linoleic acid, oleic acid, and palmitoleic acid. Differences in fatty acid composition were found among the varieties studied (Melgarejo and Artes, 2000). Fatty acid composition in the seed oil of 25 pomegranates varieties from two different regions of India showed oil content of 6.6–19.3%, most of it unsaturated fatty acids. The predominant fatty acid was linolenic acid (31.8–86.6%), followed by linoleic acid, oleic acid, stearic acid and palmitoleic acid. The varieties studied had similar but not identical fatty acid composition Parashar et al., 2010).

Significant differences were found in studies conducted with different varieties in different regions of the world, indicating that genetic background influences this trait. Nevertheless, the general structure of lipids, mainly in seed oil, is very similar (Figure 3A). The vast majority of the fatty acids are unsaturated fatty acids and punicic acid is by far the main fatty acid. Sitosterol is the most abundant phytosterol in pomegranate seed oil.

##### Composition of lipids in juice and peel

Pande and Akoh (2009) investigated the lipid profiles of six Georgia (USA) pomegranate varieties. Ripe fruits were used for the preparation of two fractions: seed tissue and non-seed tissues (peels and juice). Total lipid content in the seeds was 18.1–21.5% and 0.2–0.3% in the non-seed fraction. Punicic acid was the predominant fatty acid in the seed lipids and linoleic acid was the major fatty acid in the non-seed fraction. All the varieties had the same saturated/unsaturated fatty acids ratio of 0.1 for the seeds and 0.5 for non-seed tissues. Linoleic acid, palmitic acid, and oleic acid are most abundant in aril juice and peel and are secondary in abundance in seeds; however, punicic acid was not detected in the non-seed tissues. Pomegranate seed had a high content of α- and γ-tocopherol (167.3 and 84.6 mg/100 g, respectively). The most abundant phytosterol was β-sitosterol, ranging from 32.7 to 345.8 mg/100 g. There were more phospholipids in seeds than in aril juice and peel. Phosphatidylcholine content varied from 5.8 to 23.1 mg/100 g and phosphatidylethanolamine ranged from 10.2 to 74.2 mg/100 g in all the seed varieties. Significant differences were found between the four cultivars in their different lipid components (Pande and Akoh, 2009). Figure 3B illustrates a representative picture of the relative content of fatty acids in pomegranate non-seed fruit tissues (based on Pande and Akoh, 2009).

#### Differences During Fruit Development

Only one publication describes the changes that occur during seed development. Al-Maiman and Ahmad (2002) studied unripe, half-ripe, and fully-ripe fruits of Saudi Arabian pomegranate “Taifi.” Lipid concentration in seeds during fruit maturation was 0.2, 0.01, and 0.25%, respectively and unsaturated fatty acids constituted 81.6, 82.1, and 84.6% of all fatty acids. No significant changes were observed in lipid concentration and fatty acid classes in the seeds during fruit development (Al-Maiman and Ahmad, 2002).

#### Climate and Geographic Influence

To our best knowledge, only one study compared the content of seed oil among the same varieties under different climates. However, no study that deals with climate or geographical influence on fruit lipid composition was published. Seed oil content of several pomegranate varieties was measured for trees grown in a Mediterranean climate (Newe Ya'ar) and desert climate (Arava desert). Oil content ranged from 7.76 to 17.96 g/100 g of dried seeds, varying for the different accessions. Four accessions (P.G.114-15, P.G.116-17, P.G.128-29, and P.G.130-31) exhibited significantly higher oil contents when grown in the southern Arava compared to Newe Ya'ar. These results suggest that fruits grown in a hot dry climate may have higher oil content in their seeds (Schwartz et al., 2009).

## Secondary Metabolites

### Anthocyanins

Anthocyanins are the key color molecules of pomegranate present in various parts of the pomegranate trees, including leaves, flowers, and fruits. The pomegranate fruit is a rich source of anthocyanins and produces several derivatives of anthocyanins. These secondary metabolites accumulate in all fruit tissues and mainly in the edible part of the fruit, the arils, and in the fruit peel (Gil et al., 1995b; Hernandez et al., 1999; Tzulker et al., 2007). Six anthocyanin molecules were identified in pomegranate fruit, including mono- and di-glucosides of cyanidin (red pigments), delphinidin (purple pigments), and pelargonidin (orange pigments) (Du et al., 1975; Gil et al., 1995b). All six anthocyanin pigments were detected in pomegranate cultivars from different geographical regions, which include Israeli, Turkish, Spanish, Californian, Tunisian, Italian, and Chinese pomegranates (Gil et al., 1995a,b; Ben-Simhon et al., 2011; Turkyilmaz, 2013; Zhao et al., 2013). However, differences in the relative amounts of anthocyanins were found, depending on variety, climatic, and cultural variables (Gil et al., 1995a; Ben-Simhon et al., 2011; Borochov-Neori et al., 2011). Some unusual anthocyanin molecules were reported by Fischer et al. (2011a,b), who detected cyanidin pentoside in pomegranate peel and juice and cyanidin rutinoside and cyanidin pentoside-hexoside in the juice. Zhao et al. (2013) reported that peonidin hexoside and myricetin hexoside were detected in the peel of a dark red Chinese cultivar. These findings suggest that the pigment profile of pomegranates may be much more diverse.

The function of anthocyanin in the biology of the pomegranate tree is not yet fully understood. The tree of the “white” phenotype pomegranate varieties, which do not produce any anthocyanin (Ben-Simhon et al., 2015), is vigorous and fertile. It seems, however, that the white flowers and anthocyanin-less fruits are more susceptible to browning and radiation damages (personal communication). The accumulation of anthocyanin in young pomegranate leaves also suggests that it acts to protect the tissues from abiotic and biotic stresses during leaf development.

#### Differences Among Varieties

##### Composition of anthocyanins in the peel

In an attempt to determine the color variability among pomegranate varieties, 29 varieties that represent most of the phenotypic variability in the Israeli pomegranate collection were assayed. Total anthocyanin levels were measured for both peel and aril extracts. The content of total anthocyanins in the peel varied between 0.2 and 8.0 × 10^2^ mg/L, while the anthocyanin content in the aril juice varied between 0.2 and 3.5 × 10^2^ mg/L (Tzulker et al., 2007). The high variation that was detected in peel total anthocyanin content is also observed by the naked eye. Thus, fruit from various varieties in the Israeli collection display colors which range from purple to dark red to green (Tzulker et al., 2007; Holland and Bar-Ya'akov, 2008; Holland et al., 2009, Figure 4). Three pomegranate cultivars with different colors were also studied in China for their anthocyanin content in fruit peel and juice. Significant differences were found in anthocyanin concentration among different cultivars in their fruit peel (up to 344 mg/100 g) and in their juice (up to 364 mg/100 g) (Zhu et al., 2015).

**Figure 4:**
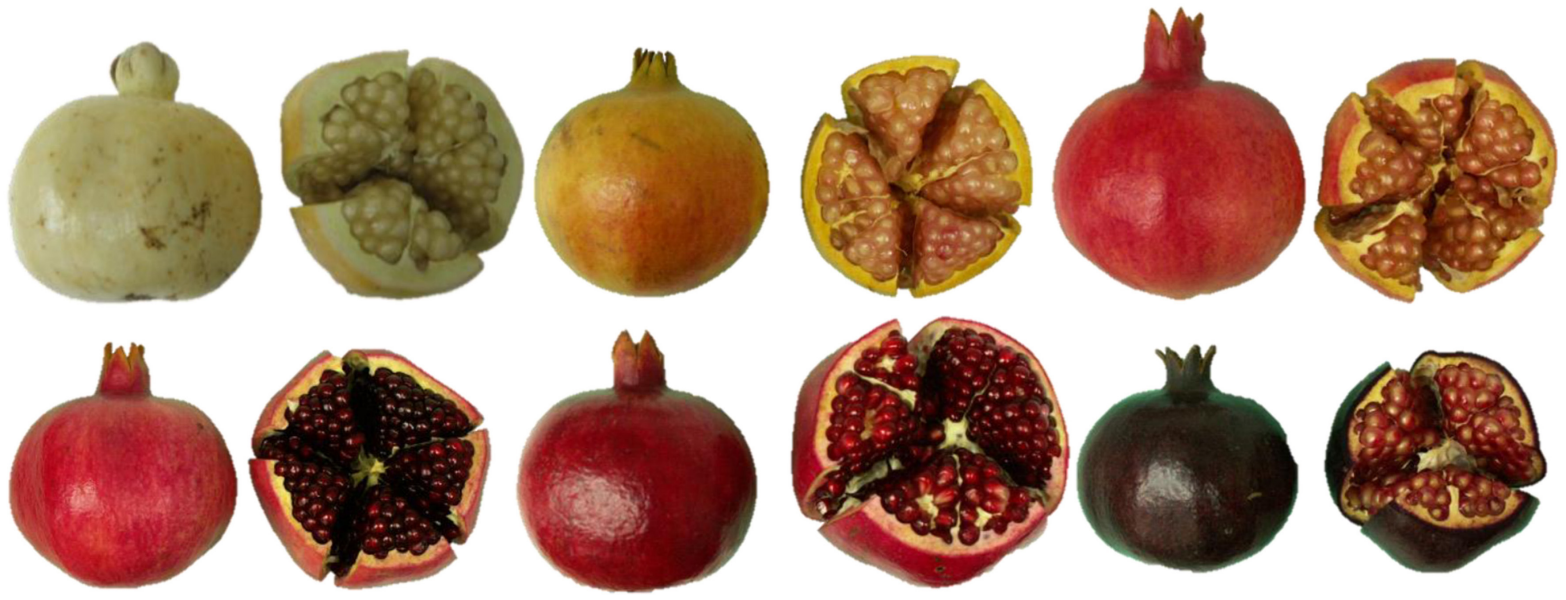
Fruit peel and arils of various varieties in the Israeli pomegranate collection display a wide range of colors.

No correlation was observed between the content of anthocyanin in the peel and in the arils. The predominant color of the fruit peel is mostly the outcome of accumulation of cyanidin derivatives. Cyanidine derivatives constitute about 85% of the anthocynins in ripened pomegranate fruit, while pelargonidin derivatives constitute about 15% in ripened fruit (Ben-Simhon et al., 2011). Only low or undetectable levels of delphinidins were usually found in the fruit skin (Du et al., 1975; Gil et al., 1995b; Ben-Simhon et al., 2011). However, in some dark red Chinese cultivars, large amounts of cyanidin mono-glycoside and delphinidin mono-glycoside (over 100 mg/100 g) were found in the skin (Zhao et al., 2013).

##### Composition of anthocyanins in the juice

While most Israeli and Mediterranean cultivars displayed negligible levels of delphinidines in their skins, delphinidins, and cyanidins were the major anthocyanins in their aril juice (Gil et al., 1995b; Seeram et al., 2006a; Borochov-Neori et al., 2011). In Israeli cultivars delphinidine derivative content could reach about 40% of the total anthocyanin content of the aril juice and cyanidines could reach about 60% of the total anthocyanins in the aril juice. Similar data was also reported for “Mollar” in Spain, where delphinidine derivatives constituted about 50% and cyanidine constituted about 45% of the aril juice anthocynins (Gil et al., 1995b). Juices from fruits of 30 varieties grown in Tunisia were studied for their anthocyanin content. The total anthocyanin content was different among varieties and ranged from 9 to 115 mg/L juice (Hasnaoui et al., 2011). Aligourchi et al. (2008) measured the amounts of total anthocyanins in the juice of 15 pomegranate varieties obtained from Yazd province in Iran. There was significant difference in total anthocyanin levels among varieties ranging from 15.0 to 252.2 mg/L juice.

From these studies of different varieties originating from several regions in the world and from many others not reported here, it is evident that there are significant quantitative and qualitative differences in the anthocyanin content of peel and juice between pomegranate varieties. These differences can be attributed to the diverse genetic background of the fruits tested.

#### Differences During Fruit Development

The differences found in the composition and quantity of anthocyanin between the peel and the arils suggest that anthocyanin accumulation in these tissues reflects differential genetic control of anthocyanin production. This assumption is further supported by the different dynamics of anthocyanin accumulation in the peel and arils during fruit development (Ben-Simhon et al., 2011; Holland and Bar-Ya'akov, 2014). This tissue- specific differential accumulation of anthocyanins is one of the main difficulties in determining the ripening time of pomegranate fruit by external phenotypic parameters.

##### Composition of anthocyanins in the juice

Kulkarni and Aradhya (2005) reported total anthocyanin content in squeezed arils of the Indian “Ganesh” at 20, 40, 60, 80, 100, 120, and 140 days after fruit set. A 100% increase in the anthocyanin concentration was observed between 20 and 80 days after fruit set. The highest concentration (138 mg/100 g) was recorded after 100 days, followed by a slight significant decrease (9.3%) up to 140 days of fruit development (Kulkarni and Aradhya, 2005). Shwartz et al. (2009) investigated changes in total anthocyanin content in the arils of two Israeli varieties during fruit maturation. The anthocyanin levels in the aril juice increased significantly during maturation in “Wonderful,” which had red aril color (from 165 to 328 mg/L), but not in “Rosh Hapered,” which had light pink aril color. Hernandez et al. (1999) studied changes in the quantity and quality of anthocyanins in the juice of five Spanish pomegranate varieties during ripening. Juice from the fruit 26–34 weeks after flower set (immature fruits to commercially mature fruits) was extracted and analyzed. Generally, there was an increase in juice pigmentation during fruit ripening, but total anthocyanin during fruit maturation differed among varieties. Three varieties did not show any change in the first 4 weeks of fruit development and then, the anthocyanin concentration increased rapidly until ripening. The two other varieties already showed an increase in anthocyanin content at very early stages. In the early fruit development stages, delphinidin di-glucoside was the main pigment, followed by cyanidin di-glucoside, while in the later stages, the mono-glucoside derivatives of cyanidin and delphinidin increased considerably. The pelargonidin derivatives were always present in small amounts. The authors found that fruits located in the north side of the trees showed an earlier increase in anthocyanin pigmentation, which was explained by lower temperatures during the night in this niche (Hernandez et al., 1999). The pattern of di-glucosides in the first stages of fruit development and mono-glucosides anthocyanin prevailing in the latter stage was also shown in another study done by Gil et al. (1995b) in Spain.

##### Composition of anthocyanins in the peel

Zhao et al. (2013) studied three Chinese cultivars of pomegranates with fruit peel colors ranging from green to dark red. The fruit skin was analyzed for anthocyanin content and composition during fruit development at 10-day intervals until full ripening. They found that cultivars' peel color and fruit developmental stage significantly influenced the profile of the anthocyanins and their content. The pigment content generally increased toward ripening and the relative amounts of the six primary anthocyanin molecules changed (Zhao et al., 2013). Shwartz et al. (2009) also studied the changes in anthocyanin content in the peel of two pomegranate cultivars. Peel color significantly changed during maturation in both varieties. Total anthocyanin was significantly correlated with color index in both accessions, indicating that the total anthocyanin content contributed significantly to the peel's skin color (Schwartz et al., 2009).

#### Climate and Geographic Influence

One of the most interesting aspects of pomegranate color from academic and practical point of view is the influence of environmental conditions on color accumulation. It is well-known that pomegranate fruit color, like that of other anthocyanin-accumulating plants, such as grapes, red orange, and roses, is sensitive to high temperatures (Lo Piero et al., 2005; Ubi et al., 2006; Mori et al., 2007; Ferrara et al., 2015). When fruits of evergreen pomegranates that can produce all year round were tested in the Arava desert in Israel during winter and summer, it was found that the content of anthocyanin in the aril juice was inversely related to the sum of heat units accumulated during fruit ripening (Borochov-Neori et al., 2009). Moreover, it was found that in addition to their effects on the content of anthocyanins, the change of season influenced the level and composition of the anthocyanin derivatives in the juice. Thus, cyanidine molecules accumulated in the hotter season and delphinidin derivatives accumulated in the cooler season (Borochov-Neori et al., 2011). It was also noticed that di-glycosidic derivatives mostly accumulated in the hot season, while mono-glycosidic derivatives were mostly accumulated during the cooler season, suggesting that di-glycosidic conjugates of anthocyanins are more stable in higher temperatures (Borochov-Neori et al., 2011). The effect of temperature on anthocyanin accumulation was also demonstrated when the content of anthocyanin in 11 different cultivars grown in the Arava desert in Israel was compared to the content of anthocyanin of the same cultivars grown accumulation in aril juice and peel was suggested (Schwartz et al., 2009).

Anthocyanin content in the arils and peel of pomegranate fruit is also sensitive to salt stress (Borochov-Neori et al., 2013). When two different pomegranate cultivars were irrigated with saline water it was found that increased salinity had a positive influence on anthocyanin accumulation in the pomegranate fruit peel (Borochov-Neori et al., 2014). The increase in anthocyanin accumulation corroborates the proposed function of anthocyanins in plant response to environmental stress conditions (Chalker-Scott, 1999; Hatier and Gould, 2009; Steyn, 2009). The magnitude of the effect of increased salinity concentrations from 1 to 6 dS^m−1^ was about 4-fold (from 20 to 80 mg/Kg) of anthocyanin for the highly colorful “Wonderful,” and about 8-fold (from 5 to 40 mg/Kg) for the pale color “SP-2.” These findings differ from those obtained for the anthocyanin in the arils (Borochov-Neori et al., 2014), where salinity had an adverse effect on anthocyanin accumulation, especially in “Wonderful.” As for exposure to different temperatures, exposure to salinity affected the level of anthocyanin derivatives. At elevated salinity levels, “Wonderful” fruit peel accumulated purple delphinidins in addition to the major pigment types, cyanidins and pelargonidins, whereas in “SP-2” the proportion of the orange color pelargonidins increased.

The significance of these data to the physiology of the fruit and trees is not yet understood. However, it showed that anthocyanin content is dynamic and depends on environmental conditions, water quality and the genetic background of the trees. This understanding is important for commercial perspectives, as it determines the choice of cultivars in different environmental conditions and geographical locations. It also influences the quality and suitability of the fruit for medical or nutritional consumption.

#### Genetics

The high variability in color of the skin and arils of pomegranate suggest a strong genetic control of anthocyanin production in pomegranate. Several expressed genes that are highly correlated with anthocyanin accumulation during fruit development were first identified by Ben-Simhon et al. (2011). These genes were initially isolated on the basis of their homology to known genes involved in the production of flavonoids and anthocyanins. They included the structural genes: *PgLDOX* (*ANS*), *PgDFR*, and *PgCHS* and the regulatory genes: *PgTTG1* (*WD40*), *PgAN1* (*BHLH*), and *PgAn2* (*Myb*). Up until now the only genes from pomegranate for which a confirmed function in anthocyanin production was reported are the genes which encode for the enzyme leucoanthocyanidin oxidase *PgLDOX* (Ben-Simhon et al., 2015) and for the *WD40* type of transcription factor *PgTTG1* (Ben-Simhon et al., 2011). The function of the pomegranate gene *PgTTG1* was shown by complementing the TTG1 mutant of arabidopsis with the pomegranate *PgTTG1* homolog (Ben-Simhon et al., 2011). In this case, the *pgTTG1* function was demonstrated for both the ability to regulate anthocyanin production and for regulating trichome formation. The function of *PgLDOX* was confirmed by identifying the site of the recessive mutation within its coding sequence located between positions 90–91 downstream of the ATG initiation codon. The mutation disrupts the gene in the anthocyanin-less pomegranate mutant. This mutation abolishes the expression of *LDOX* in all the pomegranate tissues and prevents the accumulation of anthocyanin (Ben-Simhon et al., 2015). The clear linkage of the mutation to the inability to produce anthocyanins was accomplished by genetic mapping, using segregating F2 populations for a white phenotype mutant that does not produce anthocyanins in its fruit and leaf tissues (Ben-Simhon et al., 2015). The identification of *PgLDOX* as the gene responsible for the anthocyanin-less pomegranate phenotype was supported by Zhang et al. (2009), who showed that the anthocyanin-less mutant does not express the *PgLDOX* gene. These authors cloned several additional candidate genes from white and red pomegranate cultivars related to anthocyanin synthesis and studied their expression (Zhao et al., 2015). The recent determination of the pomegranate genome and expression analysis of candidate genes combined with the accumulation of anthocyanins in flowers and the outer seed coats during development provide a more comprehensive list of putative genes involved in the anthocyanin synthesis pathway (Qin et al., 2017).

### Hydrolyzable Tannins

In addition to anthocyanins, pomegranate is also a rich source of hydrolyzable tannins. Hydrolyzable tannins are further divided into gallotannins and ellagitannins according to the phenolic groups that are esterified to the hydroxyl groups of glucose: gallic acid in gallotannins and hexahydroxydiphenic acid (HHDP) in ellagitannins. More than 60 hydrolyzable tannins have been (tentatively) identified in pomegranate, of which over 30 are reportedly present in fruit peel, aril juice, and seed (Fischer et al., 2011a; Mena et al., 2012; Ito et al., 2014; Ambigaipalan et al., 2017; Wu and Tian, 2017; Liu and Seeram, 2018) (Table 3).

**Table 3:** Hydrolyzable tannins (tentatively) identified in pomegranate fruit peel, aril juice, and seed tissues. HHDP, hexahydroxydiphenic acid; (+) reported presence; (–) presence not yet reported.

**Hydrolizable tannin**	**Fruit peel**	**Aril juice**	**Seed**
3,3′-Di-O-methylellagic acid	–	–	+
3,3′,4′-Tri-O-methylellagic acid	–	–	+
Brevifolin carboxylic acid	+	+	–
Casuarinin	+	–	–
Dehydro-galloyl-HHDP-hexoside	–	+	–
Di-HHDP-galloylglucose-pentoside	–	+	–
Digalloyl-gallagyl-hexoside	–	+	–
Digalloyl-triHHDP-diglucoside (sanguiin H10)	–	+	–
Digalloyl hexoside	+	+	+
Digalloyl-HHDP-glucoside (punigluconin)	+	–	–
Ellagic acid	+	+	+
Ellagic acid pentoside	+	+	+
Ellagic acid deoxyhexoside	+	+	+
Ellagic acid hexoside	+	+	+
Eucalbanin B	–	+	–
Eucarpanin T1	–	+	–
Gallagic acid	+	–	–
Gallagyl hexoside	+	–	–
Galloyl-HHDP-glucuronide	+	–	–
Galloyl-gallagyl-hexoside	+	–	–
Galloyl hexoside	+	+	–
Galloyl-HHDP-hexoside (Corilagin)	+	+	+
Granatin A	+	–	–
Granatin B	+	+	-
HHDP hexoside	+	+	+
Lagerstannin B	+	–	–
Lagerstannin C	+	+	–
Oenothein B	–	–	–
Pedunculagin I	+	+	–
Pedunculagin II	+	+	–
Pomegraniin A	–	+	–
Pomegraniin B	–	+	–
Punicacortein C	+	–	–
Punicalagin α	+	+	–
Punicalagin β	+	+	–
Punicalin α	+	+	–
Punicalin β	+	+	–
Tellimagrandin I	+	–	–
Trisgalloyl hexoside	+	+	–
Valoneic acid dilactone	+	+	+

In pomegranate fruit peels, punicalagin α and β isomers (designated punicalagins) are the predominant form of hydrolyzable tannins accounting for over 85% of total tannins (Seeram et al., 2005). Other major hydrolyzable tannins in fruit peels include punicalin, ellagic acid, gallagic acid, and ellagic acid glycosides. In most cases, hydrolyzable tannins are present in both fruit peel and aril juice (Table 3). There are only a few reports on hydrolyzable tannins in pomegranate seeds; interestingly, 3,3′-di-*O*-methylellagic acid and 3,3′,4′-tri-*O*-methylellagic acid have been identified in seeds, but not in fruit peels or aril juice (Table 3). It is worth noting that metabolite identification depends on the pomegranate accessions that are being analyzed. For example, a castalagin derivative and a galloyl-bis-HHDP-hexoside (casuarinin) derivative were only detectable in fruit peels of “Acide,” but not in the other three Tunisian pomegranate accessions (“Gabsi,” “Nebli,” and “Tounsi”) (Abid et al., 2017). Conversely, punicalagins, galloyl-HHDP-hexoside, galloyl-HHDP-DHHDP-hexoside (granatin B), and digalloyl-HHDP-hexoside (pedunculagin II) were present in fruit peels of all four accessions, and most abundant in “Acide” (Abid et al., 2017).

Efficient and effective metabolite extraction methods are also a key to understanding the composition and content of hydrolyzable tannins of pomegranates and their different tissues.

#### Differences Among Varieties

Although there is a wide variety of pomegranate accessions worldwide (Holland et al., 2009), only a small portion of these accessions have been analyzed in detail for hydrolyzable tannins.

##### Composition of hydrolyzable tannins in the juice

Pomegranates have traditionally been consumed for fresh aril juice; therefore, several studies focused on quantification of hydrolyzable tannins in this tissue. Aril juices of 12 commercial pomegranate varieties and 5 non-commercial varieties grown and harvested in different regions (Israel, Turkey, Spain, Iran, Tunisia, and Italy) contained 139.7–473.4 mg/L of ellagic acid and 300–810 mg/L of total phenolic acids and hydrolyzable tannins (Gómez-Caravaca et al., 2013). Ellagic acid levels in the aril juices of eight Iranian cultivars were evaluated and ranged from 7 to 160 mg/L. Interestingly, total tannins, ranging from 15 to 32 mg/100 g, showed an inverse correlation with ellagic acid concentrations in these cultivars (Mousavinejad et al., 2009).

In recent years, industrial procedures have been established that press juice from whole pomegranate fruits. Therefore, the commercial pomegranate juices contain hydrolyzable tannins from aril juice as well as other parts of the fruit. For example, the commercial juices of “Wonderful” contained 1,500–1,900 mg/L of punicalagins, about 100-fold higher than those present in aril juice (Gil et al., 2000). Similarly, punicalagins were in the range of 31–607 mg/L in aril juices, and 156–1,169 mg/L in whole fruit juices of 10 Iranian pomegranate cultivars (Akhavan et al., 2015). However, the levels of punicalagins in the aril juices of some cultivars were particularly high, e.g., the punicalagin level in the aril juice of “JPGRT” (607 mg/L) was significantly higher than those in whole fruit juices of cultivars “PSY” (156 mg/L), “VKT” (286 mg/L), “MY” (338 mg/L), “SRAB” (549 mg/L), and “TML” (569 mg/L) (Akhavan et al., 2015).

Four major hydrolyzable tannins, including punicalagins, punicalins, gallagic acid, and ellagic acid, were quantified from whole fruits and aril juices of 29 local and domesticated Israeli accessions (Tzulker et al., 2007). In addition to variations in the relative abundance of the four hydrolyzable tannins (puncalagins on the scale of 10^5^ mg/L, punicalins 10^4^ mg/L, gallic acid 10^3^ mg/L, and ellagic acid 10^2^ mg/L in whole fruit extracts), the accessions analyzed were largely different in the concentrations of each hydrolyzable tannin in whole fruits and aril juices. Furthermore, the hydrolyzable tannins were a thousand fold less concentrated in aril juices than in whole fruits (Tzulker et al., 2007).

##### Composition of hydrolyzable tannins in the peel

Pomegranate fruit peels, though inedible, contribute to hydrolyzable tannins in commercial juice products and have drawn attention for being a rich source of valuable compounds. Total phenolics in fruit peels of four Tunisian cultivars were studied. Not only varietal differences in total tannins were observed, there were also more tannins in the acetone than in the water or ethanol extracts (Abid et al., 2017).

##### Composition of hydrolyzable tannins in the seeds

In comparison with fruit peels and aril juices, hydrolyzable tannins are less abundant in seeds. Total tannins, including gallotannins, ellagic acid derivatives, and gallagyl tannins (mainly punicalagins and punicalins) were 4,792–6,894 mg/L in fruit peels of six cultivars grown in the southern United States, which were 50- to 60-fold and over 100-fold higher than those in aril juices and seeds, respectively (Pande and Akoh, 2009). Punicalagins, punicalins, gallic acid, and ellagic acid were quantified in fruit peels, aril juices, and seeds of five widely consumed pomegranate cultivars in China (Li et al., 2016). Punicalagins were found in fruit peels in the range of 61.75–125.23 mg/g dry weight. In all of the cultivars analyzed, fruit peels contained more punicalagins and punicalins than aril juices did, while these hydrolyzable tannins were not detected in seeds (Li et al., 2016).

Although hydrolyzable tannin composition and content cannot be directly compared among different studies due to the different extraction and quantification methods they employed, it can be concluded that hydrolyzable tannins vary in different pomegranate accessions grown in the same region, suggesting genetic contributions to hydrolyzable tannins. On the other hand, variations in hydrolyzable tannins were also observed for the same cultivar, such as “Wonderful,” when grown in multiple locations in the world. This phenomenon can be due to the many landraces of “Wonderful” and additionally suggests that climate and cultivation have an effect on hydrolyzable tannins.

#### Differences During Fruit Development

Several studies have compared hydrolyzable tannin profiles in developing pomegranate fruits. However, the fruit developmental stages were defined by different standards, such as days after fruit set/full bloom, physico-chemical properties, or physiological attributes of the fruit (Fawole and Opara, 2013b). Developing fruits of two cultivars, “Wonderful” and “Rosh Hapered,” grown in Israel were collected during a span of 8 or 10 weeks (Shwartz et al., 2009). Three major hydrolyzable tannins, gallagic acid, punicalin isomers (designated punicalins), and punicalagins, were quantified in water extracts of the developing fruits. All three hydrolyzable tannins showed decreased accumulation in developing fruit peels in both cultivars (Shwartz et al., 2009). Fruits of “Ruby” grown in South Africa were harvested at five stages according to days after full bloom (Fawole and Opara, 2013a). Total hydrolyzable tannins in aril juice declined during the progression of fruit maturation, and were accompanied by decreases in ellagic acid and gallic acid (Fawole and Opara, 2013a).

Relative amounts of hydrolyzable tannins in fruit peel, aril juice, and seed of developing pomegranate fruits were also investigated. Fruits of the Chinese cultivar “Taishanhong” were harvested at 10-day intervals for nine collections. Unicalagins, ellagic acid, and gallic acid were higher in fruit peel than aril juice and in seed; all three metabolites showed decreased accumulation in the three tissues during fruit development (Han et al., 2015). When quantified by absorption of the methanolic extracts at 550 nm, total hydrolyzable tannins gradually decreased in fruit peels at low, low-medium, medium, and medium-high (corresponding too early to late fruit development) stages of the Spanish cultivar “Mollar de Elche.” In contrast, they were not detectable in aril juice at all stages. In seeds total hydrolizable tannins increased at medium and then decreased at medium-high stages (Fernandes et al., 2015b).

Overall, despite the differences in the genetic background, growth conditions, harvesting scheme, and extraction and quantification methods, there is a consistent trend of decreasing hydrolyzable tannin accumulation in fruit peels, aril juice, and seed through pomegranate fruit development.

#### Climate and Geographic Influence

To understand the impact of growth environment on hydrolyzable tannin profiles, fruit peel and aril juice hydrolyzable tannins were compared for 11 accessions grown in the Mediterranean or desert climate in Israel (Schwartz et al., 2009). Mediterranean climate promoted high levels of hydrolyzable tannins in aril juice in most of the accessions evaluated; in contrast, desert climate had a positive impact on hydrolyzable tannins in fruit peels (Schwartz et al., 2009). It was reported that the sweet/sour phenotype and environment interactions had the most influence (54.4%) on total tannin variations in aril juice of 10 commercial cultivars grown in four different regions in China, followed by the growth environment (45.6%). There were negative correlations of overall average temperature with total polyphenol, total tannin, and punicalagin concentrations. The sweet/sour phenotype only accounted for 0.06% of the variations in tannins among different cultivars (Li et al., 2015).

The quality of aril juice under deficit (i.e., reduced) irrigation was investigated in Spain (Mena et al., 2013). Three water regimes were applied to pomegranate trees at 75% evapotranspiration (ET_o_, non-stressed control), 43% ET_o_ (moderate water stress), and 12% ET_o_ (severe water stress). Water stress drastically decreased punicalagins, causing 30 and 70% reduction in moderate and sever stresses, respectively, in aril juices of fruits harvested from the corresponding trees (Mena et al., 2013). This study provided valuable information on the implications of water stress on the hydrolyzable tannin metabolism and the nutritional value of aril juice.

#### Genetics

To allow functional assessment of hydrolyzable tannin metabolic and regulatory genes *in planta*, a pomegranate hairy root culture system was established that produces a substantial amount of hydrolyzable tannins and is easily transformable (Ono et al., 2012). The hairy root culture system has been successfully utilized for genetic characterization of candidate hydrolyzable tannin biosynthetic genes in pomegranate, such as *PgUGT84A23* and *PgUGT84A24* involved in β-glucogallin production (Ono et al., 2016). Together with the recently published reference genomes (Qin et al., 2017; Yuan et al., 2017), pomegranate hairy roots hold great potential for functional genomics in the hydrolyzable tannin pathway.

## Summary

In this review, we have made an effort to summarize the most updated data on primary metabolites and on the most notable secondary metabolites of pomegranate fruit. Along with this effort, it was important for us to reflect the variability of metabolite content and composition and its dependence on the genetic background and environmental conditions. Finally, we present a summary of the main metabolites identified in pomegranate fruit peel, aril juice, and seed tissues (Table 4).

**Table 4:** The main metabolites identified in pomegranate fruit peel, aril juice, and seed tissues.

**Tissue**	**Sugars**	**Organic acids**	**Amino acids**	**Proteins**	**Fatty acids**	**Anthocyanins**	**Hydrolyzable tannins**
Peel	Glucose Fructose Or Xylose Arabinose	Citric acid	Glutamate Glycine Aspartate	Unknown	Linoleic acid Palmitic acid Oleic acid	Cyanidin Pelargonidin	Punicalagin
Aril juice	Glucose Fructose	Citric acid	Glutamine Serine Aspartate Or Proline Serine Alanine	Unknown	Linoleic acid Palmitic acid Oleic acid	Cyanidin Pelargonidin Delphinidin	Ellagic acid
Seed	Unknown	Unknown	Glutamate Arginine Aspartate	Globulins Albumins	Punicic acid	Unknown	Unknown

## Author Contributions

The manuscript was written by DH, IB-Y, LT, and RA. All authors assisted in writing all the chapters. DH is the corresponding author and focused on anthocynins, lipids, proteins, and amino acids. LT focused on hydrolizable tannins. RA focused on sugars and organic acids. IB focused on lipids, proteins, amino acids together with DH.

### Conflict of Interest Statement

The authors declare that the research was conducted in the absence of any commercial or financial relationships that could be construed as a potential conflict of interest.
